# Antimicrobial and antioxidant activities of the extracts and compounds from the leaves of *Psorospermum aurantiacum* Engl. and *Hypericum lanceolatum* Lam.

**DOI:** 10.1186/1472-6882-12-136

**Published:** 2012-08-24

**Authors:** Patricia D Tchakam, Paul K Lunga, Théodora K Kowa, Antoine Honoré N Lonfouo, Hippolyte K Wabo, Léon A Tapondjou, Pierre Tane, Jules-Roger Kuiate

**Affiliations:** 1 Laboratory of Microbiology and Antimicrobial Substances, Department of Biochemistry, Faculty of Science, University of Dschang, P.O. Box 67, Dschang, Cameroon; 2 Department of Biochemistry, Faculty of Science, University of Yaounde 1, P.O. Box 812, Yaounde, Cameroon; 3 Department of Chemistry, Faculty of Science, University of Dschang, P.O. Box 67, Dschang, Cameroon

## Abstract

**Background:**

*Psorospermun aurantiacum* and *Hypericum lanceolatum* are plants locally used in Cameroon and other parts of Africa for the treatment of gastrointestinal and urinary tract infections, skin infections, venereal diseases, gastrointestinal disorder, infertility, epilepsy as well as microbial infections. The present study was designed in order to investigate the *in vitro* antimicrobial and radical scavenging activities of the extracts and isolated compounds from the leaves of these plants.

**Methods:**

The plant extract was prepared by maceration in ethyl acetate and methanol and fractionated by column chromatography. The structures of isolated compounds were elucidated by spectroscopic analyses in conjunction with literature data. The broth microdilution method was used to evaluate the *in vitro* antimicrobial activity against bacteria, yeasts and dermatophytes. The antioxidant potentials of the extracts and their isolated compounds were evaluated using the DPPH radical scavenging method.

**Results:**

Five known compounds: physcion (**1**), 1,8-dihydroxy-3-geranyloxy-6-methylanthraquinone (**2**), kenganthranol B (**3**), vismiaquinone (**4**), and octacosanol (**5**) were isolated from the leaves of *P*. *aurantiacum* while six compounds including friedelin (**6**), betulinic acid (**7**), 2,2’,5,6’-tetrahydroxybenzophenone (**8**), allanxanthone A (**9**), 1,3,6- trihydroxyxanthone (**10**) and isogarcinol (**11**) were isolated from *H. lanceolatum.* Compound 8 and 4 exhibited the highest antibacterial and antifungal activities with MIC ranges of 2–8 μg/ml and 4–32 μg/ml respectively. *P. aurantiacum* crude extract (Rsa50 = 6.359 ± 0.101) showed greater radical scavenging activity compared with *H. lanceolatum* extract (Rsa50 = 30.996 ± 0.879). Compound **11** showed the highest radical scavenging activity (RSa_50_ = 1.012 ± 0.247) among the isolated compounds, comparable to that of L-arscobic acid (RSa50 = 0.0809 ± 0.045).

**Conclusions:**

The experimental findings show that the ethyl acetate and methanol extracts and isolated compounds from *P. aurantiacum* and *H. lanceolatum* stem bark possess significant antimicrobial and antioxidant activities justifying the use of these plants in traditional medicine, which may be developed as phytomedicines.

## Background

During the last 20 years, it has been reported that human infections are increasing at an alarming rate, especially in tropical and subtropical developing countries [1]. This is partly due to the indiscriminate use of antimicrobial drugs and the development of microbial resistance to some of the synthetic drugs [2]. Resistance to most antibiotics occurs through the aegis of extremely efficient enzymes, efflux proteins and other transport systems that often are highly specialized towards specific antibiotic molecules [3]. The fact that microorganisms nowadays tend to develop resistance towards drugs, coupled to the undesirable side effects of certain antibiotics offer considerable potentials for the development of new effective antimicrobial agents; medicinal plants being a prolific source. Various plant extracts possess bacteriostatic and bactericidal effects due to secondary metabolites they contain, namely alkaloids, tannins, flavonoids, and phenolic compounds. Most of these secondary metabolites other than possessing antimicrobial potential, can also act as potent antioxidants [4].

*Psorospermun aurantiacum* and *Hypericum lanceolatum* are trees, both belonging to the family of Guttiferae and are generally found in mountainous areas [5]. In Cameroon, *Psorospermun aurantiacum* appears in the North-West and West Regions, where the decoction of the leaves is used to treat gastrointestinal and urinary tract infections. Combined with other plant extracts, the stem bark is used to treat epilepsy. The fruits of this plant have been recently investigated for their phytochemical constituents [6]. *Hypericum lanceolatum* on the other hand, occurs on mountains in the Western Region of Cameroon, and is used for the treatment of skin infections, venereal diseases, gastrointestinal disorder, tumours, infertility and epilepsy [5,7]. Higher plants like those from the Guttiferae family are rich sources of antimicrobial phenolic secondary metabolites which are able to act as reducing agents, hydrogen donors, and singlet oxygen quenchers [8-11].

Several antifungal [1], antibacterial [12,13], anticancer [14,15] and antiviral [16] compounds have been isolated from *Hypericum* genus. In the present paper, we report the isolation of constituents from *Hypericum lanceolatum* and *Psorospermun aurantiacum* together with some related antimicrobial and antioxidant activities of these constituents and the crude extracts.

## Methods

### Plant material

The leaves of *Psorospermum aurantiacum* and *Hypericum lanceolatum* were separately collected in May 2009 at Mount Bamboutos, West Region of Cameroon. Authentification of the plants was done by Mr. Nana Victor at the Cameroon National Herbarium where voucher specimens were kept under the reference numbers of 52651 HNC and 32356 HNC respectively.

### Extraction, fractionation and isolation

The air-dried and powdered leaves of *P. aurantiacum* (2.60 kg) and of *H. lanceolatum* (2.00 kg) were extracted respectively with EtOAc and MeOH at room temperature (3 × 12 l, 72 h) to obtain corresponding crude extracts of 77 g and 60 g after evaporation under vacuum. The two solvents were selected based on their extraction yields from preliminary extractions studies. Part of the crude extract of *P. aurantiacum* (67 g) was subjected to silica gel column chromatography, eluted with gradients of *n*-hexane-CH2Cl2 (10:0, 9:1, 8:2, 1:1; 0:10) and CH2Cl2-MeOH (9:1, 8:2, 1:1; 0:10). Twenty three (23) fractions of 500 ml each were collected and grouped on the basis of their thin layer chromatography (TLC) profiles into five major fractions F1-F5 (F1: 1–4; F2: 5–8; F3: 9–14; F4: 15–20 and F5: 21–23). These fractions were submitted to repeated column chromatography over silica gel and purified by preparative TLC. Octacosanol (10 mg) was obtained from fraction F1. Fractions F2 and F3 afforded 1,8-dihydroxy-3-geranyloxy-6- methylanthraquinone (8 mg) and vismiaquinone (11 mg) respectively. Fraction F4 afforded kenganthranol B (10 mg) and physcion (25 mg).

Fifty-five grams (55 g) of the crude extract of *H. lanceolatum* was subjected to silica gel column chromatography eluted with gradients of *n*-hexane-EtOAc (10:0, 9:1, 8:2, 1:1; 0:10) and EtOAc-MeOH (9:1, 8:2, 1:1; 0:10). Thirty-eight fractions of 500 ml each were collected and combined on the basis of TLC analysis to afford five main fractions (A–E). Fraction A contained mostly fatty materials and was not further investigated. Fraction B was further separated by silica gel column chromatography eluted with *n*-hexane-EtOAc to give white needles of friedelin (14 mg). Betulinic acid (1.2 g) was purified from fraction C by recrystallization in MeOH. Fraction D was submitted to a silica gel column chromatography eluted with *n*-hexane-EtOAc to afford three sub-fractions (D1-D3). Sub-fraction D1 was further purified by Sephadex LH-20 using CH2Cl2-MeOH (1:1) to give 2,2’,5,6’-tetrahydroxybenzophenone (30 mg). Repeated column chromatography of sub-fraction D2 on silica gel using increasing mixtures of *n*-hexane-EtOAc yielded allanxanthone A (16 mg) and 1,3,6-trihydroxyxanthone (7 mg). Isogarcinol (18 mg) was purified from sub- fraction D3 by recrystallization in *n-*hexane.

### Identification of isolated compounds

The structural elucidation of compounds was done on the basis of physical data and spectroscopic analysis, including 1D and 2D NMR and direct comparison of the data with those reported in the literature. Melting points were determined with a Reichert microscope and are uncorrected. UV spectra were measured with a UV-210 PC, UV/Vis scanning spectrophotometer (Analytikjena). IR spectra were recorded on a Shimadzu FTIR-8400S spectrophotometer with KBr. EIMS (ionization voltage 70 eV) and HREIMS mass spectra were recorded on Jeol JMS AX-500 and AX-700 or a 6890 N Network GC System/5975 Inert XL Mass Selective Detector GCMS spectrometers. ^1^H NMR (500 MHz) and ^13^C NMR (125 MHz) spectra were recorded in CDCl_3_ or CDCl_3_-CD_3_OD using a Bruker Avance 500 MHz spectrometer. Silica gel 60 (70–230; Merck; Darmstadt, Germany) was used for column chromatography. Precoated Silica gel 60 Kieselgel F_254_ plates (0.2 mm thick) were used for TLC, and the spots were detected with ultraviolet (UV) illumination and by spraying with 50% (v/v) H_2_SO_4_, followed by heating at 100°C. Merck Silica gel 60 F_254_ was used for preparative thin layer chromatography.

### Antimicrobial assay

#### Microorganisms and culture media

The microorganisms used in this study were obtained from the American Type Culture Collection (ATCC), “Ecole Nationale Vétérinaire d’Alfort” (E), “Institut Pasteur de Paris” (IP) and “Centre Pasteur de Yaoundé”. They included five bacteria species (*Klebsiella pneumonia* ATCC 13883, *Pseudomonas aeruginosa* ATCC 27853, *Shigella flexneri, Salmonella typhi* ATCC 6539 *and Enterococcus faecalis* ATCC 10541) and six fungal species (*Candida lusitaniae* ATCC 200950, *Candida krusei* ATCC 6258, *Candida albicans* ATCC 2091, *Cryptococcus neoformans* IP 95026, *Trichophyton ajelloi*, *Trichophyton rubrum*). The culture medium, Sabouraud Dextrose Agar (SDA, Conda), was used for subculturing during minimum fungicidal concentration determination. Sabouraud Dextrose Broth (SDB, Conda) was used for the determination of minimum inhibitory and fungicidal concentrations.

#### Preparation of microbial inocula

The inocula of bacteria and yeasts were prepared from 24 h old broth cultures. The absorbance was read at 600 nm and adjusted with sterile physiological solution to match that of a 0.5 McFarland standard solution. From the prepared microbial solutions, other dilutions with sterile physiological solution were prepared to give a final concentration of 10^6^ colony- forming units (CFU) per milliliter for bacteria and 2 × 10^5^ spores per milliliter for yeasts.

Conidia suspensions of dermatophyte species were prepared after separation and filtration of conidia from 15 days old cultures. The number of conidia was determined using a spectrophotometer and adjusted with sterile physiological solution to an absorbance of 0.60 at 450 nm corresponding to a final concentration of 1 × 10^5^ spores/ml [17].

#### Determination of minimum inhibitory concentration (MIC) and minimum microbicidal concentrations (MMC)

The MICs of the crude extract and isolated compounds were determined by the broth microdilution method in 96-well micro-titre plates as described by Zgoda et Poter (2001) [18]. The 96-well plates were prepared by dispensing into each well 100 μl of Mueller Hinton broth for bacteria and Sabouraud Dextrose broth for fungi. The test substances were initially prepared in 10% DMSO in broth medium at 4096 μg/ml for the extracts and 512 μg/ml for compounds or 50 μg/ml for the reference antibiotics. A volume of 100 μl of each test sample was added into the first wells of the micro-titre plate (whose wells were previously loaded with 100 μl of broth medium). Serial two-fold dilutions of the test samples were made and 100 μl of inoculum standardized at 10^6^ CFU/ml for bacteria, 2.5 × 10^5^ CFU/ml for yeasts (at 600 nm, Jenway 6105 UV/Vis spectrophotometer- 50 Hz/60 Hz) [19] and 1 × 10^5^ spores/ml for dermatophytes (at 450 nm) were then added into each well. This gave final concentration ranges of 1024 to 0.5 μg/ml for the extracts, 128 to 0.0625 μg/ml for isolated compounds and 12.50 to 0.006 μg/ml for reference substances. The plates were sealed with parafilm, then agitated with a plate shaker to mix their contents and incubated at 35°C for 24 h for bacteria, 48 h for yeast and at 28°C for 5 days for dermatophytes.

For bacteria, MICs were determined upon addition of 50 μl (0.2 mg/ml) *p*-iodonitrotetrazolium chloride (INT, Sigma-Aldrich, South Africa). Viable bacteria reduced the yellow dye to a pink colour. For yeasts and dermatophytes, MICs were determined by visualising the turbidity of the wells. The MIC corresponded to the lowest well concentration where no colour/turbidity change was observed, indicating no growth of microorganism. The MBC or MFC was determined by transferring 50 μl aliquots of the clear wells into 150 μl of freshly prepared broth medium and incubating at 35°C for 24 h (bacteria), 48 h (yeasts) and at 28°C for 5 days (dermatophytes). The MBC or MFC was regarded as the lowest concentration of test sample which did not produce a colour/turbidity change as above, indicating no microbial growth. All tests were performed in triplicates. Ciprofloxacin for bacteria, nystatin for yeast and griseofulvin for dermatophytes were used as positives controls.

### Antioxidant activity

Radical scavenging activity (RSa) of the extract and pure compounds from *P. aurantiacum* was determined using the stable free radical 2,2’-diphenylpicrylhydrazyl (DPPH) as described by Ghomi et al. (2008) [20]. Two-fold serial dilution was made from a 625 μg/ml stock solution of each sample to obtain concentration ranges of 625 to 78.12 μg/ml. A quantity of (100 μl) of each test solution was mixed with 900 μl of a freshly prepared DPPH-methanol solution (20 mg/l) and allowed to stand for 30 min in the dark at room temperature. The optical densities of the resulting solutions were read at 517 nm using a Jenway UV/vis 6105 spectrophotometer. L-ascorbic acid was used as positive control. Radical scavenging activity (RSa) was calculated as follows:

$$\text{\%RSa}=\frac{\text{Absorbance}\;\text{of}\;\text{DPPH}-\text{Absorbance}\;\text{of}\;\text{sample}}{\text{Absorbance}\;\text{of}\;\text{DPPH}}\times 100$$

Sample concentration providing 50% radical scavenging activity (RSa50) was calculated from the graph of %RSa as a function of log [sample concentration].

### Statistical analysis

The data on antioxidant activity were subjected to the one-way analysis of variance (ANOVA) and results were expressed (where appropriate) as mean ± standard deviation. Differences between means of samples were compared using Duncan’s multiple range tests at *P <* 0.05.

## Results and discussion

**Figure 1:**
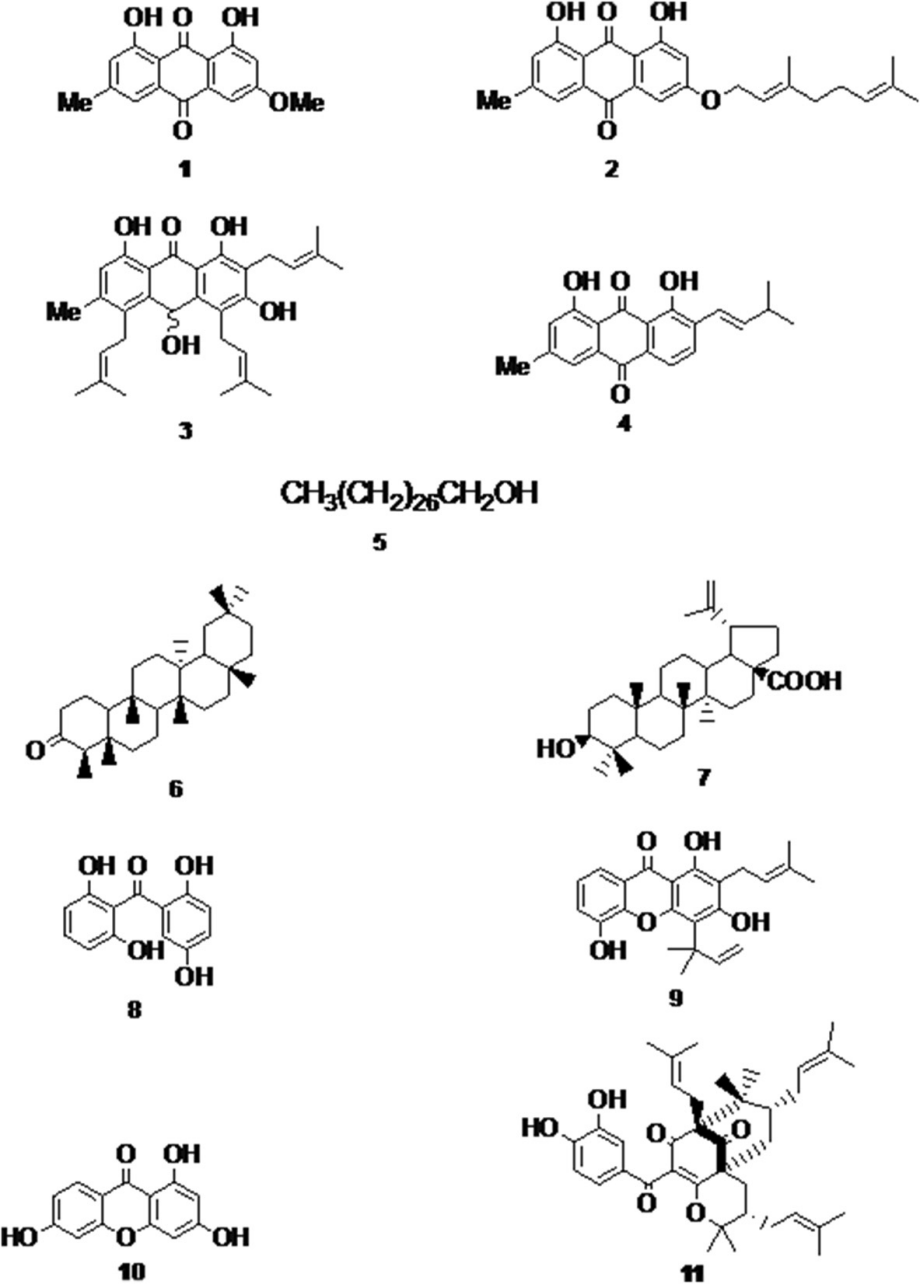
**Chemical structures of physcion (1), 1,8 dihydroxy-3-geranyloxy-6-methylanthraquinone (2), kenganthranol B (3), vismiaquinone (4), octacosanol (5) from *P. aurantiacum *and friedelin (6); Betulinic acid (7); 2,2’,5,6’-tetrahydroxybenzophenone (8); allanxanthone A (9); 1,3,6-trihydroxyxanthone (10); Isogarcinol (11) from *H. lanceolatum***.

The following known compounds: physcion (**1**) [21], 1,8-dihydroxy-3-geranyloxy-6- methylanthraquinone (**2**) [22], kenganthranol B (**3**) [6], vismiaquinone (**4**) [23], octacosanol (**5**) [24] (Figure 1) were isolated and identified in the leaves of *P*. *aurantiacum*. From the MeOH leaf- extract of *H. lanceolatum* friedelin (**6**) [11], betulinic acid (**7**) [25], 2,2’,5,6’- tetrahydroxybenzophenone (**8**) [7], allanxanthone A (**9**) [26], 1,3,6-trihydroxyxanthone (**10**) [27] and isogarcinol (**11**) [28] were isolated and identified (Figure 1). The compounds isolated in the present study were formerly isolated from other plants and diverse activities were demonstrated. *H. lanceolatum* extract and its isolated compounds have been proven to possess anti-malarial properties [29].

The antimicrobial properties of the extracts and isolated compounds of *P. aurantiacum* and *H. lanceolatum* are presented in Table 1. In general, the crude extract of *H. lanceolatum* presented a wide range of activity and was more active than that of *P. aurantiacum*. Isogarcinol (**11**) and octacosanol (**5**) were the most active compounds with relatively high antibacterial activities, particularly on *S. flexneri, K. pneumoniae* and *S. typhi* while physcion (**1**) showed virtually no antimicrobial activity at the tested concentrations on bacteria. This is interesting in the perspective of developing more potent antibacterial agents especially when regarding the global diseases burden of the susceptible microorganisms [30,31]. Isogarcinol, a benzophenone derivative is known to possess cytotoxic effect [32].

**Table 1:** Minimum inhibitory concentration (MIC)/Minimum bactericidal or fungicidal concentration (MBC or MFC) of tests substances (μg/ml)

		***P. aurantiacum***						***H. lanceolatum***				
	**Parameters**	**Extract**^**a**^	**1**	**2**	**3**	**4**	**5**	**Extract**^**b**^	**7**	**10**	**11**	**RD**
Bacteria												
*K. pneumoniae*	MIC	256	-	16	128	8	4	64	125	4	16	1
	MBC	1024	-	32	128	16	-	1024	-	*4*	*16*	*1*
	*MBC*/*MIC*	*4*	*-*	*2*	*1*	*2*	-	16	-	1	1	1
*S. flexneri*	MIC	1024	-	64	-	16	8	64	2	8	16	0.50
	MBC	>1024	-	64	-	16	8	1024	2	8	16	0.50
	*MBC*/*MIC*	nd	-	1	-	1	1	16	1	1	1	1
*P. aeruginosa*	MIC	1024	-	32	-	8	-	64	-	8	16	1
	MBC	>1024	-	64	-	16	-	1024	-	8	16	1
	*MBC*/*MIC*	nd	-	2	-	2	-	16	-	1	1	1
*S. typhi*	MIC	-	-	-	-	-	2	32	2	2	4	0.50
	MBC	nd	nd	nd	nd	nd	2	1024	2	2	4	0.50
	*MBC*/*MIC*	nd	nd	nd	nd	nd	1	32	1	1	1	1
*E. faecalis*	MIC	-	-	-	-	nd	32	64	32	8	32	0.50
	MBC	nd	nd	nd	nd	nd	32	1024	32	8	32	0.50
	*MBC*/*MIC*	nd	nd	nd	nd	nd	1	16	1	1	1	1
Fungi/ Yeats												
*C. albicans*	MIC	1024	-	-	-	32	-	512	-	32	64	2
	MBC	>1024	nd	nd	nd	32	-	1024	-	32	64	2
	*MBC*/*MIC*	nd	nd	nd	nd	1	-	2	-	1	1	1
*C. lusitaniae*	MIC	256	16	8	8	4	-	-	-	-	-	1
	MBC	256	16	32	16	8	-	nd	-	nd	nd	1
	*MBC*/*MIC*	1	1	4	2	2		nd	-	nd	nd	1
*C. neoformans*	MIC	1024	16	-	-	32	125	512	-	32	64	0.5
	MBC	1024	16	nd	nd	64	125	1024	-	32	64	1
	*MBC*/*MIC*	1	1	nd	nd	2	1	2	-	1	1	2
*C. krusei*	MIC	1024	64	-	-	32	-	256	-	64	64	2
	MBC	1024	64	nd	nd	32	-	1024	-	64	64	2
	*MBC*/*MIC*	1	1	nd	nd	1	-	4	-	1	1	1
Fungi/Dermatophytes												
*T. ajelloi*	MIC	1024	16	16	4	4	4	512	4	32	64	4
	MBC	1024	16	16	4	4	4	1024	4	32	64	4
	*MBC*/*MIC*	1	1	1	1	1	1	2	1	1	1	1
*T. rubrum*	MIC	1024	32	128	16	8	32	256	64	8	32	4
	MBC	1024	32	128	16	8	32	1024	64	8	32	4
	*MBC*/*MIC*	1	1	1	1	1	1	4	1	1	1	1

Extract^a^ : EtOAc extract of the leaves of *P. aurantiacum*; Extract^b^ : MeOH extract of the leaves of *H. lanceolatum*; nd: not determined; -: not active at the concentration up to 128 μg/ml for compounds and 1024 μg/ml for extracts; RD (Reference drug) ciprofloxacin for bacteria, nystatin for yeast and griseofulvin for dermatophytes.

In general, the antifungal activities of isolated compounds were relatively lower than their antibacterial activities. However, kenganthranol B (**3**), vismiaquinone (**6**), octacosanol (**7**) and betulinic acid (**9**) showed relatively high antidermatophytic activity particularly on *T. ajelloi*. Mbaveng et al. (2008) [33] previously demonstrated the antimicrobial properties of vismiaquinone isolated from *Vismia guineensis* and this corroborate the present study. The results obtained with kenganthranol B corroborate those of Kouam et al. (2006) [34] who reported that kenganthranol E possess no antibacterial activity against *Bacillus megaterium*, *Escherichia coli*, *Chlorella fusca* and *Microbotryum violaceum*. Despite the relatively low antimicrobial activity of kenganthranol B, it has demonstrated, together with some of its isomers, to possess alpha-glucosidase inhibitory activity [34]. All the isolated compounds were generally less active than the reference antibiotics. The ratio MBC/MIC was generally ≤ 4 with respect to all the microorganisms studied, indicative of a possible bactericidal nature of the test samples [35].

**Figure 2:**
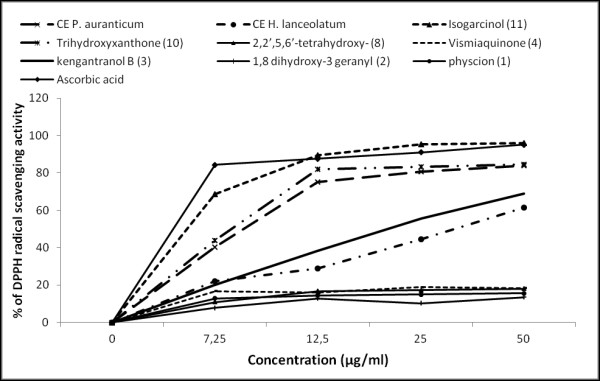
**DPPH radical scavenging activities of crude extracts and compounds from *P. aurantiacum *and *H. lanceolatum***.

Many microbial infections lead to the production of highly reactive molecules from the metabolism of oxygen that can cause extensive damage to cells and tissues [36]. The extracts and isolated compounds exhibited differential radical scavenging activity against the stable DPPH free radical (Table 2 and Figure 2). At high concentrations (12.5 -50 μg/ml) the activity of isogarcinol was comparable to that of L-ascorbic acid, the reference molecule. Although isogarcinol was isolated from *H. lanceolatum*, the crude extract of this plant showed a relatively low activity compared to that of *P. aurantiacum*. This may either be due to low concentration of this compound in the extract or to an antagonistic effect with other constituents of the extract. Isogarcinol is an isomeric form of garcinol, known to possess high antioxidant activity among many other properties [37].

**Table 2:** **Radical Scavenging activity 50 values (μg) of the methanol extracts and compounds from the leaves of *H. Lanceolatum *and *P. aurantiacum***

**Test substance**	**Rsa50 (μg)**
*H. lanceolatum* extract	30.99 ± 0.87e *P.*
*aurantiacum* extract	6.35 ± 0.10b,c
Isogarcinol	1.01 ± 0.24b
1,3,6-trihydroxyxanthone	4.73 ± 0.80b
Kenganthranol B	21.93 ± 5.98d
2,2’,5,6’-tetrahydroxybenzophenone	na
Physcion	na
1,8-dihydroxy-3-geranyloxy-6-methylanthraquinone	na
Vismiaquinone	na
L-ascorbic acid	0.089 ± 0.05a

Values with the same letter superscripts are not significantly different using Waller Duncan test at p ≤ 0.05. na: not active.

## Conclusion

The present findings support the ethno-pharmacological exploitation of these plants in the treatment of microbial infections and hold great perspective in the development of alternative antimicrobial and antioxidant agents from isogarcinol.

## Competing interests

Authors declare that they do not have any competing interests.

## Authors’ contributions

PDT was the field investigator and drafted the manuscript. PKL revised the manuscript. TKK ANHL, LAT and PT carried out the isolation and characterization of compounds. HKW and JRK designed the study, supervised the work and corrected the manuscript. All authors read and approved the final manuscript.

## Pre-publication history

The pre-publication history for this paper can be accessed here:

http://www.biomedcentral.com/1472-6882/12/136/prepub
